# [Corrigendum] Exosomal microRNA‑4516, microRNA‑203 and SFRP1 are potential biomarkers of acute myocardial infarction

**DOI:** 10.3892/mmr.2024.13289

**Published:** 2024-07-10

**Authors:** Peng Liu, Shuya Wang, Kaiyuan Li, Yang Yang, Yilong Man, Fengli Du, Lei Wang, Jing Tian, Guohai Su

Mol Med Rep 27: 124, 2023; DOI: 10.3892/mmr.2023.13010

Subsequently to the publication of the above article, the authors have realized that, in Fig. 1A, the incorrect image was uploaded to show the ultrastructure of exos isolated from plasma and examined using transmission electron microscopy (essentially, the image in question had already appeared in an article published by the same research group in *Journal of Cellular and Molecular Medicine*). In addition, the ‘+’ and ‘-’ signs for the ‘Cell lysis’ experiments shown underneath the gels in Fig. 1B were incorporated the wrong way around.

The revised version of Fig. 1, showing the correct image in Fig. 1A and the correct labels in Fig. 1B, is shown below. Note that the errors made in assembling this figure did not have a major impact on either the results or the conclusions reported in this paper. The authors are grateful to the Editor of *Molecular Medicine Reports* for allowing them this opportunity to publish a corrigendum, and apologize to the readership of the Journal for any inconvenience caused.

## Figures and Tables

**Figure 1.**
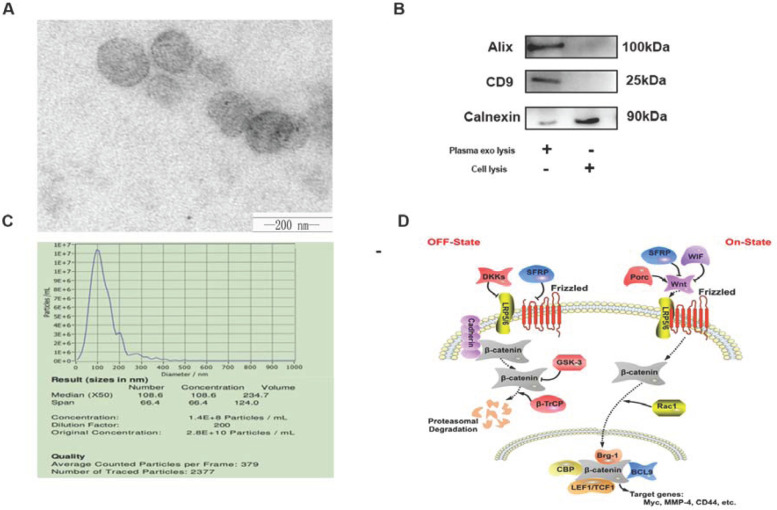
Successful isolation of exos from plasma. (A) Ultrastructure of exos isolated from plasma was examined using transmission electron microscopy. Scale bar=200 nm. (B) The expression of exo markers, Alix and CD9, and the negative marker, calnexin, assessed using western blotting. (C) Size distribution of exos using particle size analysis. (D) SFRP1 serves an inhibitory role in the Wnt/β-catenin signaling pathway. Exo, exosome; SFRP1, secretory frizzled-related protein 1.

